# Greater willingness to reduce microplastics consumption in Mexico than in Spain supports the importance of legislation on the use of plastics

**DOI:** 10.3389/fpsyg.2022.1027336

**Published:** 2023-01-11

**Authors:** Eva Garcia-Vazquez, Cristina Garcia-Ael, Maritza Librada Caceres Mesa, Noemi Rodriguez, Eduardo Dopico

**Affiliations:** ^1^Department of Functional Biology, University of Oviedo, Oviedo, Spain; ^2^Faculty of Psychology, Universidad Nacional de Educación a Distancia UNED, Madrid, Spain; ^3^Department of Educational Sciences, Universidad Autónoma del Estado de Hidalgo, Pachuca, Mexico; ^4^Department of Education Sciences, University of Oviedo, Oviedo, Spain

**Keywords:** consumer awareness, Mexico, microplastics, Spain, pro-environmental behavior, microplastics risk awareness

## Abstract

**Introduction:**

Microplastics (MP) threaten all organisms worldwide. MP are produced directly as microbeads in cosmetics and hygiene products, or indirectly from breakage of larger plastics. The control of MP requires consumers' engagement to refuse products containing microbeads.

**Methods:**

We conducted a survey on 572 university students from Mexico and Spain, two countries where microbeads are not banned yet. More strict laws for plastic control areenforced in Mexico than in Spain.

**Results:**

Controlling for age and education, despite knowing less about MP, Mexicans checked for microbeads on product labels more frequently than Spaniards, and desired to reduce MP consumption more. A stronger correlation between individual awareness and willingness of MP control was found for Mexican than for Spanish students.

**Discussion:**

Perhaps more strict legislation against plastics creates an environment favorable to MP control. Unclear statement of microbeads on labels was the main reason for not checking microbead contents; environmental education and a stricter control of plastics and MP were identified as necessary policy changes in the two countries. Corporation engagement on clearer product labeling is also suggested.

## 1. Introduction

The problem of microplastics (hereafter referred to as MP) has a global dimension. Water and MP are the sole elements common to all ecosystems nowadays as well as in life occurring worldwide, from the atmosphere to the poles and from the highest mountains to abyssal plains (Peng et al., 2017; Chen et al., 2020; Mishra et al., 2021). MP are defined as plastics smaller than 5 mm (Thompson et al., 2004), and represent an emerging pollutant whose removal from ecosystems is not easy; efficient cleanup technology is currently being investigated (Magalhães et al., 2020; Vivekanand et al., 2021).

MP are harmful for all organisms that have been studied to date. MP cause adverse effects with different natures, including physiological and neurological damage, growth retardation, oxidative damage, among others (Barboza et al., 2020; Gola et al., 2021). From fish consumption only, humans intake ~840 MP/year (Barboza et al., 2020). We also breathe in a considerable amount of MP, especially microfibers (Gasperi et al., 2018); ~48,000 MP particles per day are taken in by inhalation (Wieland et al., 2022). MP are composed of toxic monomers that cause inflammation, alteration of immune function, reproductive toxicity, mutagenesis, and cancer in humans (Gasperi et al., 2018; Prata et al., 2020).

Primary MP (i.e., those of this small size that are produced) are included in many products such as cosmetics and personal care products, cleaning agents, paint, and coatings (Van Wezel et al., 2016). Microbeads are added to cosmetics for exfoliation, face and body cleansing, as well as to toothpaste; both rinse-off and leave-on products (Anagnosti et al., 2021). Due to their known risk and their accumulation in the environment and organisms (Rochman et al., 2015), non-degradable plastic microbeads are banned in personal care products in eleven countries in North America, Asia, Europe, and New Zealand; further bans are also currently proposed in six other countries, and microbeads are being phased out in three more (Dauvergne, 2018; Lam et al., 2018; Anagnosti et al., 2021). This implies that, in the majority of countries worldwide, microbead bans are not yet in place. Moreover, other types of primary MP such as polyester glitters coated with metal are widely employed in cosmetics, textiles, and household applications, and these are generally overlooked (Yurtsever, 2019) and not explicitly considered in the bans. Industrial abrasives or laundry detergents that significantly contribute to MP pollution (e.g., Rochman et al., 2019) are also not included in the bans.

In a global market dominated by supply and demand, citizens may help to stop MP production by taking individual actions as consumers; especially in countries where microbead bans are not yet planned. Evidently, regularly checking for the presence of microbeads on product labels, thus avoiding the purchase of MP-bearing goods is a first, easy behavior that can be adopted, instead using eco-friendly MP-free products. In this study, we will explore whether current pro-environmental legislation may promote these simple behaviors in societies where MP bans have not yet been implemented. Policy and legislation have been described as effective top-down means to promote pro-environmental practices in different countries and cultures. Examples of this are the reduction of poisoning practices promoted by the awareness of environmental legislation in Kenya (Didarali et al., 2022), the pro-environmental engagement of Australian employees following the introduction of corporate pro-environmental policies (Albrecht et al., 2021), and the increased environmental concern of Chinese citizens living in cities with more sustainability policies in place (Liu et al., 2018).

Country-level policies on the use of plastics may help the residents of that country to develop a generic attitude toward plastic derivatives such as MP. Legislation regarding plastic consumption differs greatly between countries (Adeyanju et al., 2021). For example, in Mexico, total bans on single-use plastics have been in force in several states since 2020. Mexico City was one of world's pioneer megacities to adopt bans on this type of plastic, with legislation introduced in 2019 to ban plastic bags and products containing MP, which was then extended to all single-use plastics in January 2021 (Decreto Congreso Ciudad de Mexico I Legislatura, 25 June 2019; available online at http://legismex.mty.itesm.mx/estados/ley-df/DF-L-ResSol-Ref2019_06.pdf). In contrast, only levies are applied in Spain, specifically to plastic bags (Royal Decree 293/2018, 18 May 2018; available online at: https://www.boe.es/eli/es/rd/2018/05/18/293). The level of support for plastic bans and international treaties against plastic pollution is higher in Mexico than in Spain (IPSOS/Plastic Free; July 2022). In this study, we investigate whether this is extended to MP.

## 2. Literature review

Knowledge of MP is the first step required for people to become aware of MP impact and—perhaps—change their behavior. The majority of consumers are not conscious of acquiring products containing MP (Henderson and Green, 2020; Ojinnaka and Aw, 2020). Yan et al. (2020) found that people are unaware of MP due to their invisibility. Moreover, because of their small size, MP are not easily detectable in the environment; thus, they have only relatively recently been considered as an emerging contaminant (Katsnelson, 2015). Since they are difficult to observe with the naked eye, most people are aware of them thanks to the media (Henderson and Green, 2020). For this reason, knowledge occupies a central position in the psychosocial landscape of behaviors associated with MP emissions (Garcia-Vazquez and Garcia-Ael, 2021). According to the Theory of Reasoned Action, where knowledge is essential to change behavior intention (Ajzen and Fishbein, 1980), information concerning MP increases awareness and intention of behavior change, as discussed in different studies (Chang, 2015; Cammalieri et al., 2020).

Although for other issues such as climate change the perception of risk predisposes people to act pro-environmentally (e.g., Bradley et al., 2020), Deng et al. (2020) showed that knowledge alone (not concern) was sufficient to increase the intention to reduce MP emissions. Those that are aware of MP will tend to reduce them, perhaps because they are clearly unnatural (Anderson et al., 2016). Even when unaware of their risks, one understands that one should not eat, breath, or live with MP. However, this issue is not completely clear, since in Korea, it is the risk perception (influenced by knowledge) that significantly affects pro-environmental behavior intention toward MP (Yoon et al., 2021). Similarly, the concern has influenced the willingness to pay for MP control in Norway (Abate et al., 2020). More studies are required to present this aspect in a clearer manner.

Another factor influencing behaviors regarding MP is the country or culture. For example, for similar levels of knowledge, the willingness to pay for MP-free products is higher in Portugal than in Germany and Norway (Misund et al., 2020). However, few studies compare attitudes toward MP among different countries. A worldwide understanding of the knowledge regarding awareness and behavior toward MP is still scarce, with some regions, such as African and American countries, clearly understudied (Garcia-Vazquez and Garcia-Ael, 2021).

Finally, socio-demographic factors such as age, gender, and education have been shown to influence attitudes and behaviors toward MP, although the different directions are not always clear. Educated people are more aware of MP (Ojinnaka and Aw, 2020). Gender and age show contradictory effects depending on the study. Abate et al. (2020) found Norwegian males to be less concerned about MP; however, they would pay more to control them. In contrast, Chinese females presented a stronger intention to reduce MP emissions than males (Deng et al., 2020). Younger people are more aware of MP in Belgium (Herweyers et al., 2020), but less so in Portugal (Soares et al., 2021). Clearly, more studies are required to understand how socio-demographic factors influence consumers' relationship with MP.

## 3. Objectives and research hypotheses

The specific objective of this study was to determine the differences between Mexico and Spain in terms of the behavior and behavioral intentions regarding MP. Mexico applies stricter plastic control measures than Spain; however, specific laws on MP are not in force therein yet. The research hypotheses were:

1) From the difference between Mexico and Spain in relation to the treatment of plastics, we expect that Mexicans avoid products containing MP (Hypothesis i) and possess a greater will to control MP (Hypothesis ii) than Spaniards.
2) Living in an environment that makes Mexicans more conscious of plastic issues, the correlation between individual awareness and willingness to control MP will be stronger in Mexico than in Spain (Hypothesis iii).

## 4. Methods

### 4.1. Ethics considerations

The competent Committee of Research Ethics of Asturias Principality approved this study, with the following reference number: CEImPA:2021.116. The participants were informed about the objective of the study, about their right to withdraw from the study at any moment, and they signed an informed consent. We followed the principles of the Declaration of Helsinki, adhering to the European code of conduct for research integrity (All European Academies, 2017).

### 4.2. Questionnaire employed

The questionnaire gathered information about the following issues: socio-demographic data, knowledge of MP, awareness of MP risk (environmental, social, and health), actual behavior regarding checking MP on commercial labels, and intended behavior of reducing the consumption of products containing MP and using eco-friendly products instead. We have collected socio-demographic data for factors that, according to the scientific literature, can influence knowledge and attitudes toward MP: age, gender, and education level. To identify possible methods to control MP, we asked for the main reasons to not check for microbeads in commercial products, and for policies to control MP.

The questionnaire was based on those created by Deng et al. (2020) and Yoon et al. (2021) that were tested and validated in China, and China, Japan, Korea, and USA, respectively. Questions on socio-demographic issues, MP knowledge, and policies for MP control were adapted from Deng et al. (2020) and those on MP risk awareness and behavioral intention from Yoon et al. (2021).

The first version of the questionnaire was examined by a panel of experts (*N* = 6). Their suggestions were introduced in the definitive questionnaire, which was re-examined and approved by the expert panel.

The questions were analyzed and their respective coding and scoring are summarized in Supplementary Table 1. Section A is devoted to socio-demographic information. It includes country (laws on plastics are different in Mexico and Spain), gender, age, and education level (A1–A4). Section B contains two questions on the actual behavior of checking for MP (frequency, and reasons for not checking for MP). Section C refers to knowledge about MP. Item C.1 is self-declared knowledge of MP. In the items C.3, C.4, and C.5 (multiple answers possible) all answers offered are true, based on the literature (e.g., (Rochman et al., 2015, 2019; Van Wezel et al., 2016; Gasperi et al., 2018; Prata et al., 2020). Thus, the actual knowledge about MP sources (C.3), environmental sinks or sites of MP accumulation (C.4), and ways in which MP can enter the human body (C.5), is the sum of the respective items marked (marked = 1, unmarked = 0). Item C.6 asks about the policies considered good to control MP. Finally, Section D contains a 7-point scale, with three items measuring the awareness of MP risks (items D.1–D.3), and two the intentions to adopt pro-environmental behaviors (items D.4 and D.5).

The questionnaire was prepared in two versions (English and Spanish) and provided according to the native language of the participants (Spanish). The English version of the questionnaire was produced by blind back-translation (Jackson et al., 1983).

### 4.3. Sampling methodology

A digital version of the questionnaire was created for online self-administration. Researchers passed the link to university students by email, with a brief message about the aim of the study (research on microplastic perceptions), its anonymous nature, and use exclusively for research. The respondents were thanked for their participation and were politely asked to pass the link to the online questionnaire to their acquaintances (snowball methodology, see Valerio et al., 2016). Before accessing the questionnaire, participants were informed about the project, authors, and policy for anonymous data treatment, and signed the informed consent.

The time estimated to complete the questionnaire was 10–15 min. Incomplete questionnaires with < 80% items completed were not considered.

### 4.4. Data analysis

#### 4.4.1. Variable scoring

Different types of variables were considered in this study. Socio-demographic variables (section A) were scored as explained in Supplementary Table 1. For correlation and regression analysis, dummies were employed for gender (0 = man, 1 = woman) and country (1 = Mexico, 0 = Spain, according to the strictness of the laws on plastics). In these analyses, non-binary respondents were excluded due to their small number.

For the declared knowledge (item C.1: “Have you heard about MP before this survey?”), a dummy was employed (0 = no, 1 = yes). The actual knowledge of MP was measured as the mean number of the sources (item C.3), sinks (item C.4), and ways in which MP can enter the human body (item C.5) identified.

In section D, a score of 1 indicates extremely unlikely/total disagreement and 7 extremely likely/total agreement. The MP risk awareness was determined as the mean of the social, health, and environmental risks perceived (items D.1, D.2, and D.3, respectively).

#### 4.4.2. Statistics

The Content Validity Index (CVI; proportion of experts rating an item as quite relevant or relevant; values over 0.78 are considered acceptable) was calculated to assess the validity of the questionnaire (Polit et al., 2007). The reliability of the variable measures, based on combined items, in this survey was determined by employing Cronbach's α, which is considered to be a good reliability index (Raykov and Marcoulides, 2017). Cronbach's α values over or around 0.8 are considered sufficient to meet reliability for applied research (Cho, 2020).

Measurement Invariance (MI) was generally checked to assess the cross-cultural equivalency of latent psychological variables measured from a number of items, often through Confirmatory Factor Analysis (CFA) (Milfont and Fischer, 2010; Hu et al., 2019). As described above, in the present study, the majority of variables are measured directly from only one item of the questionnaire, i.e., are not latent variables; thus, the model cannot be tested using CFA. Although factorial analysis was not possible, as a proxy, we explored MI, focusing on the only variables measured from several items: MP risk awareness (three items). Mardia's multivariate kurtosis and skewness tests did not meet the normality assumption for this variable; the Doornik and Hansen test result was *Ep* = 376.3 with *p* < < 0.001. Given the lack of multivariate normality, we employed RMSEA and χ^2^/df as measures of model fit, and conducted partial invariance testing (e.g., Steenkamp and Baumgartner, 1998) using JASP (JASP Team, 2022).

To test Hypotheses (i) and (ii) we employed multiple multivariate regression analysis to determine which independent variables significantly predicted the dependent variables: behavior and intended behavior in this study. Bonferroni correction was applied for multiple comparisons. For pairwise comparisons, differences in means were estimated from *t*- tests, and differences between medians from Mann–Whitney tests. To test Hypothesis (iii) ANOVA analysis was employed to compare the regressions of Mexican and Spanish MP reduction willingness, with a positive interaction represented by a higher slope in the Mexican sample.

Differences between samples in terms of the distribution of qualitative variables (obstacles to MP control, policies suggested) were tested by employing contingency statistics: Chi-square of contingency, with Cramer's V utilized to estimate the size effect. The free software PAST version 4.09 (Hammer et al., 2001) was employed.

## 5. Results

### 5.1. Questionnaire and survey data

The CVI obtained for this questionnaire from the expert panel was 0.96, showing a high degree of experts' agreement regarding the content validity of the final version. The raw results of the survey (*n* = 956, after removal of questionnaires with < 80% items completed) are publicly available in the Mendeley Data repository, under Garcia-Vazquez et al. (2022).

For this study we chose only university students due to the significant effect of education level as a predictor of pro-environmental behavior intention regarding MP, as found in previous studies (Ojinnaka and Aw, 2020). University students were also chosen due to the influence of age (Herweyers et al., 2020; Soares et al., 2021). In our samples, the factors “education level” and “age” were controlled, and were remarkably similar across groups (Table 1). The number of students that completed the questionnaire was *n* = 254 from Spain and *n* = 318 from Mexico.

**Table 1:** Descriptive data for the samples analyzed in this survey: The number of respondents, mean age (SD in parenthesis), mean education level (four indicates undergraduate and five graduate or above), and the proportion of females and non-binary persons, by country.

	**Mexico**	**Spain**
*N*	318	254
% females	66.7	72.8
% non-binary	0.9	0.4
Mean education	3.8 (0.55)	3.91 (0.63)
Age range	18–31	18–33
Individualism	30	51

Score of individualism/collectivism (higher values indicate individualism) assigned to each country by Hofstede et al. (2010).

The internal consistency was measured based on Cronbach's α values; 0.94 for items measuring MP risk awareness. This value is >0.80; thus, the construct can be considered reliable (Cho, 2020). Analysis of this latent variable presented a very robust MI (RMSEA = 0.001; χ^2^/d.f. = 0.095). The χ^2^/df value was < 5; from this, and the reliable Cronbach's values, we decided to retain all the items in this construct.

### 5.2. Effect of the country on the behavior regarding microplastics

Means and standard deviations of the variables measured per country are shown in Supplementary Table 2. The proportion of Mexican students who declared that they had heard about MP before this survey was 35.3%, significantly lower than the 67.9% of Spanish students with self-declared knowledge of MP (χ^2^ = 74, 1 d.f., *p* < < 0.001). Pairwise correlations between the willingness to control MP consumption and to buy eco-friendly products were positively and significantly correlated in Mexico (Table 2, below diagonal) and Spain (Table 2, above diagonal). In contrast, the behavior of checking for microbeads was not positively correlated with any of the other variables, and was negatively correlated with the intention to control MP consumption in Mexico (Table 2). Upon performing multivariate multiple regression analyses on the remaining variables, it was found that gender did not significantly predict the dependent variables (F_3, 565_ = 1.67, *p* = 0.17, >0.05, n.s.].

**Table 2:** Pairwise correlations between the behavioral variables analyzed in Mexican (below diagonal) and Spanish (above diagonal) student samples.

	**Check for microbeads**	**Reduce MP consumption**	**Buy eco-friendly products**
Check for microbeads		−0.018 n.s.	0.089 n.s.
Reduce MP consumption	−0.123^*^		0.827^***^
Buy eco-friendly products	−0.081 n.s.	0.889^***^	

Pearson's r-values and their significance are given, as ^*^p < 0.05 and ^***^p < 0.001.

**Figure 1:**
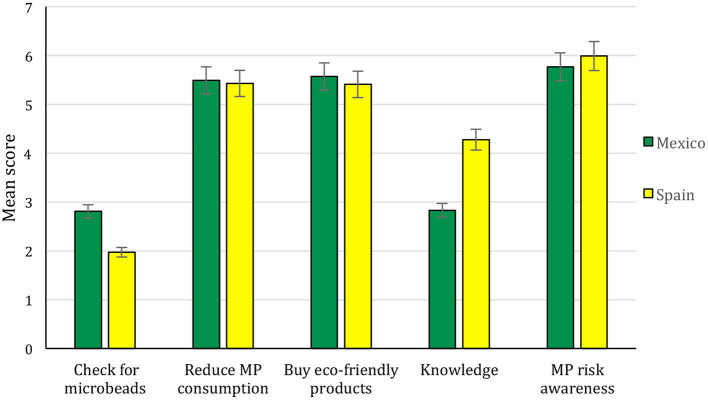
Means of Mexican and Spanish students for the variables “Checking microbeads,” “Intention to reduce microplastics consumption,” “Intention to buy eco-friendly products,” “Knowledge about microplastics,” and “Microplastics risk awareness.” Standard errors indicated.

Confirming Hypotheses (i) and (ii), the country was a highly significant predictor of the three dependent variables (*F*_3, 565_ = 17.61, *p* < < 0.001). Student samples from Mexico and Spain were significantly different to each other in terms of the three behavioral variables examined (Figure 1). Mexican students checked for microbeads more frequently (*t* = 7.33, *p* < 0.001), intended to reduce MP consumption (*t* = 2.29, *p* = 0.02), and bought eco-friendly products more than Spanish students (*t* = 2.99, *p* = 0.003), even if they knew less about MP than Spaniards (*t* = −9.7, *p* < 0.001 for actual knowledge, consistent with the self-declared knowledge in each country). The country did not significantly predict MP risk awareness in this analysis (*t* = −1.58, *p* = 0.12, n.s.).

### 5.3. Awareness and knowledge as predictors of MP control behavior in Mexico and Spain

**Figure 2:**
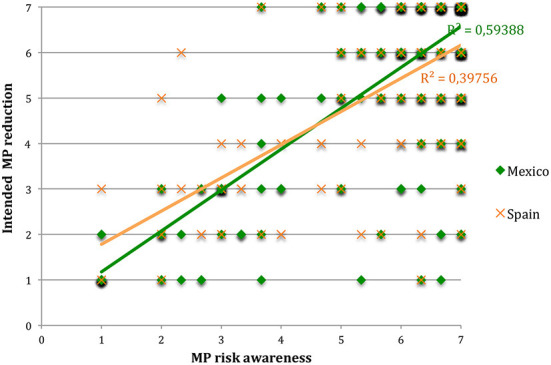
The willingness to reduce microplastics (MP) consumption vs. awareness of microplastics risk scores. Regression fits are presented in green for Mexico and orange for Spain, with their *r*^2^-values indicated.

The results showed that, controlling for the other variables, MP risk awareness significantly predicted the intention to control MP consumption in Mexico and Spain (with regression equations y = 0.899x + 0.278 and y = 0.73 + 1.05, and r^2^ = 0.59 and 0.39, respectively; both with *p* < 0.001). Confirming Hypothesis (iii), the correlation with risk awareness was higher in Mexico than in Spain (Figure 2), with a significant interaction [country] × [awareness] (*F* = 23.88, *p* = 0.003). This suggests that the awareness is significant for both countries but more significant for Mexico.

### 5.4. Obstacles and recommended policies for MP control

**Figure 3:**
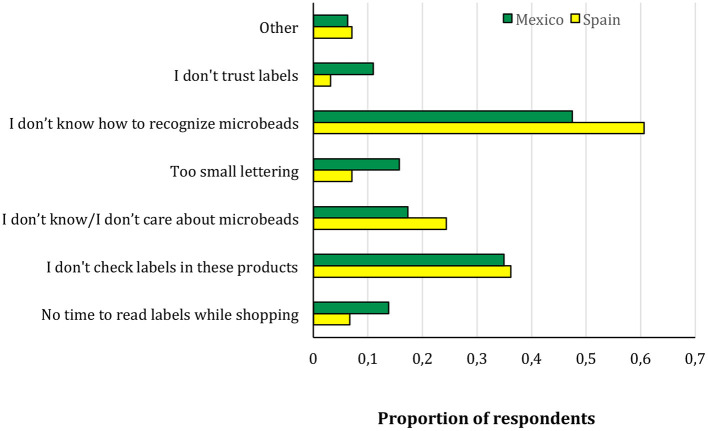
Reasons stated for not checking microbeads in the student groups surveyed in this study. Results are presented as the proportion of respondents that alleged each reason, by country. The total sum is higher than 100% because multiple answers were allowed.

The reasons for not checking for microbeads and the recommended policies for the control of MP were also analyzed in the two groups of students. Significant differences between Mexican and Spanish students were found for the alleged reasons for not checking for microbeads (χ^2^ = 38.7, 7 d.f., *p* < 0.001, Cramer's V = 0.20), with mistrust of labels, small lettering, and no time to read labels while shopping more frequent in Mexico, while not caring about microbeads and not able to identify microbeads on the labels were more frequent in Spain (Figure 3). Not recognizing microbeads from labels was the most frequent reason in both countries.

**Figure 4:**
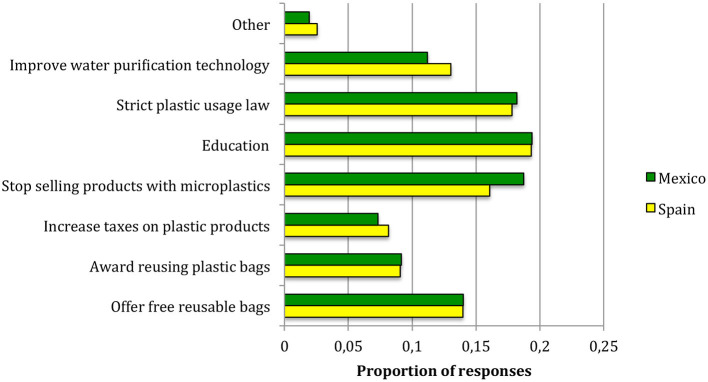
Policies recommended for the control of microplastics in the samples surveyed in this study. Results are presented as the proportion of responses that chose each policy, by country.

Regarding the recommended policies (Figure 4), there were no significant differences between the two countries (χ^2^ = 4.28, 7 d.f., *p* = 0.75 and Cramer's V = 0.05). It is worth noting that in Figure 4, the sum of percentages is 100% because, although multiple answers were allowed in these questions, we considered the proportion of responses that chose each policy over the total number of responses, by country. Policies recommended by the respondents for better control of MP were education (more than 60% of respondents chose this option, 15% greater than the total number of responses), stricter laws for plastic usage, and stop selling products with MP—which is the same as microbead bans, chosen by more than 50% of respondents and also 15% greater than the total number of responses. Offering free reusable bags and improving technology for water treatment were next (over 30% of respondents). Other options such as taxes or awards for reusing plastic bags were less supported (Figure 4).

## 6. Discussion

### 6.1. General discussion of the results

The results of this study provide novel evidence of cross-cultural differences and similarities regarding MP. For the first time, psychosocial drivers of behaviors and behavioral intentions toward MP were determined in Mexico and Spain, two countries with no microbead bans at a national level (Anagnosti et al., 2021), but with different legislation on plastics. The level of declared and actual knowledge of MP was clearly superior in Spain than in Mexico, where participants were able to identify less sources, sinks, and ways in which MP can enter the human body than Spanish participants. Notwithstanding this, Mexicans checked for microbeads more often than Spaniards did, confirming Hypothesis (i). They also declared a higher willingness to control MP according to Hypothesis (ii). The paradox of a low knowledge but a high concern in consuming and a high willingness to control MP is not so rare in the specific field of MP. In the USA, Chang (2015) found that the majority of respondents in their study were unaware of MP presence in personal care products; however, after being informed, they refused to consume those products again. Belgians would intend to buy preventive devices for microfibers when they perceive an environmental benefit, even if they know little about MP (Herweyers et al., 2020). In our study, the majority of those students hearing about MP for the first time when completing the questionnaire were from Mexico, a country already sensitized to plastics. It seems logical that they would be concerned and would reject MP, because they are clearly unnatural and unnecessary (Anderson et al., 2016).

Unlike knowledge, the awareness of risks seemed to play a more important role in our study for determining behavioral intentions. Abate et al. (2020) in Norway and Yoon et al. (2021) in Korea found that concern significantly predicted the intention to support MP control measures. Our results align with these authors since awareness of MP risks significantly predicted the willingness to control MP in both countries. Moreover, the association between awareness and MP control intention was stronger in Mexico than in Spain, according to Hypothesis (iii) and to their respective support of plastic bans (IPSOS/Plastic Free, 2022).

The difference between countries in the practice and intention of pro-environmental behaviors could be attributed to stricter legislation on plastics in Mexico, combined with other cultural traits, as we will explain below. Although individuals may exhibit oppositional behavior when top-down approaches to environmental conservation are applied (Schultz, 2011; Linklater et al., 2019), this did not seem to occur in our study. Mexicans are most likely more conscious of the threat of plastics than Spaniards, as reflected in their high support of plastic bans and international treaties (IPSOS/Plastic Free July 2022). Checking for plastics (microbeads or any other type) in personal care products is logical in a country where the legislation is stricter, and the society is in favor of plastic bans.

Together with a higher awareness of the threats of plastic, collectivism in Mexico could also explain the differences between countries. In the same vein as Sreen et al. (2018), it is possible that people in Mexico showcase more collectivistic values when making decisions regarding MP. In collectivist cultures, consumers tend to engage in behaviors that benefit society as a whole, even though these behaviors may be detrimental to them. For example, Moon et al. (2008) found that consumers in a collectivist culture were willing to pay more for products beneficial to society as a whole than those in an individualistic culture.

Although the results of this study support the departure hypotheses, they do not exactly fit into the classic Theory of Reasoned Action that is generally applied to pro-environmental behaviors (Ajzen and Fishbein, 1980), where behavioral intention is predominant and predicts actual behavior. In our study it seems that the adoption of a real behavior against MP pollution is disconnected from the behavior intention, as suggested by the non-significant or even negative correlations of checking for microbeads with intentions to reduce the consumption of goods containing MP in both countries. However, this cannot be confirmed from the results of the present study, in which the intention to check for microbeads, although implicit in the two behavioral intentions, was not explicitly posed. Those who check for microbeads on labels (to avoid products containing them) do not need to declare further intentions to do it. A similar result was found by Kim et al. (2018) in the University of Washington regarding initiatives to reduce carbon emissions: for those that were already taking pro-environmental actions, campaigns to increase environmental awareness had small effect. Likewise, in their study on university students, Cammalieri et al. (2020) found that informing about MP significantly increased awareness in less-informed students, but not so much in those that were already aware of the problem.

### 6.2. Limitations of the research

This study has some limitations; the first of which was the use of only one questionnaire version, with items presented in a given order (Dopico et al., 2022). Although the results were solidly supported by statistics, doubts about the possible effect of locating questions concerning behavior intentions at the end of the questionnaire persist. Perhaps these questions somewhat influenced the answers of those unaware of MP; however, if they were located at the beginning, it is possible that they would be unable to answer them.

From a methodological point of view, a limitation of this study was the absence of Measurement Invariance (MI) calculations for the whole model. Partial invariance testing may be not enough to ensure accurate cross-culture comparisons (Steinmetz, 2018). In the present study the majority of variables were measured from only one item; CFA is based on latent variables measured from multiple items; thus, it was not the best choice for our data. For the latent variable of MP risk awareness, the model was apparently acceptable, but the remaining variables were not analyzed. Although Welzel et al. (2021) showed cases of cultural constructs with high predictive power that do not fit MI, the results may be thus considered with caution.

Finally, this study was conducted in university student samples. Although this served to control for age and education, doubt remains regarding the representativeness of these samples of the whole society in both countries. More studies on general population samples are necessary to confirm the results of this work.

## 7. Practical implications

This study has implications for several aspects of societal support for MP control. From the low level of knowledge about MP found in some sectors of this study, measures to increase the public knowledge about this environmental problem should be taken. In our study the most frequently proposed solution was education (Figure 4). Similar recommendations (e.g., Charitou et al., 2021) are identified by the vast majority of authors working on MP and could be considered commonplace for different collectives and societies. These recommendations could be implemented at different levels. Focusing on European countries, Charitou et al. (2021) recommended more publicity for European directives, as well as integration of the topic of MP in formal education programs. We fully adhere to this recommendation and expand it to other countries such as Mexico. Increasing the knowledge on MP is especially important in countries with no microbead bans because it will contribute not only to reducing MP pollution but also to reducing the demand for those types of products. Information campaigns should be tailored by country and working sectors.

There is an urgent need to implement microbead bans and legal measures against plastics, and these two policies were proposed by the majority of respondents in our study (Figure 4). Top-down approaches could be adopted to control MP; they may reinforce attitudes favorable to plastic control, thus increasing the intention to consume MP-free products.

Here, we found significant differences between countries in terms of the awareness of MP and the actual behavior to control them. These countries have different social norms and cultures. As suggested by Garcia-Vazquez and Garcia-Ael (2021), as well as other authors Ojinnaka and Aw (2020), intercultural aspects of the psychosocial issues surrounding MP mitigation should be further explored to better determine the scale of intervention designs.

Some actions to change consumption habits regarding MP could be very simple, warranting the respect of consumers' rights. Corporations that sell hygiene and cosmetic products play important roles. They could facilitate informed consumer choice using simple practicalities, such as ensuring that information about MP on product labels is visible and understandable. In our study, the main obstacles for checking for MP on labels were unclear or illegible information concerning microbeads on the labels (Figure 3). A clearer, legible display of microbead content should appear on the labels of personal care products and cleansers.

## 8. Conclusions

In this study, Mexican respondents checked for microbeads in personal care products more than Spanish respondents and manifested a higher willingness to control MP consumption. Individual awareness was more strongly correlated with the willingness to control MP in Mexico than in Spain. Legislation favorable to the control of plastic reduction in Mexico, together with a collectivist culture, may explain this.

In this study, one of the main barriers to checking for MP was unclear information on labels. Corporations should improve the label design for personal care products and cleaners.

The participants in this study identified education as the main policy to be applied for the control of MP; therefore, campaigns that provide information on products containing MP and the widespread accumulation of this pollutant in ecosystems and organisms may help to reduce MP consumption. MP pollution risks and their global impact could be also introduced to information campaigns to increase their effect.

## Data availability statement

The original contributions presented in the study are included in the article/Supplementary material, further inquiries can be directed to the corresponding author.

## Ethics statement

The studies involving human participants were reviewed and approved by the competent Committee of Research Ethics of Asturias Principality with the reference CEImPA:2021.116. The patients/participants provided their written informed consent to participate in this study.

## Author contributions

EG-V: conceptualization, methodology, investigation, data curation, software, and writing—original draft preparation. CG-A: supervision, conceptualization, investigation, and writing—original draft preparation. NR, MM, and ED: investigation and writing—reviewing and editing. All authors contributed to the article and approved the submitted version.
