# Supplementary material for: Greater willingness to reduce microplastics consumption in Mexico than in Spain supports the importance of legislation on the use of plastics
Source: Front Psychol. 2023 Jan 11;13:1027336. doi: 10.3389/fpsyg.2022.1027336 (PMC9875725; doi:10.3389/fpsyg.2022.1027336)
Supplement: Supplementary file 1 [file Table_1.pdf]

Supplementary Table 1. Questionnaire analyzed in this study including question codes and scoring.

A.1 Gender 0 Male 1 Female 2 Non binary/Other	
A.2 Age: 1 18-30 2 30-40 3 40-50 4 50-60 5 Above 60	
A.3 Education level: 1 Junior high school or lower 2 Senior high school 3 Vocational college 4 undergraduate 5 Graduate or above	
A.4 Country (open question)	
B.1 How often do you check the microbeads content in the labels of cleansers and personal care products? 5 Every time 4 Usually 3 Sometimes 2 Seldom 1 Never	
B.2 Please choose the reason for not checking microbeads on product labels (multiple choice)	
	B.2.1 No time to read labels while shopping
	B.2.2 No check labels of this type of products
	B.2.3 I don’t know/I don’t care about microbeads
	B.2.4 Lettering on labels is too small
	B.2.5 I don’t know how to recognize microbeads in the labels
	B.2.6 I do check labels regularly
	B.2.7 I don't trust labels
	B.2.8 Other
C.1 Have you heard about microplastics before this survey? Yes = 1 No = 0 Not sure = blank	
C.3 Which of the following items do you think are the sources of microplastics? (multiple choice)	
	C.3.1 Tyres
	C.3.2 Synthetic textiles
	C.3.3 Road markings
	C.3.4 Ship paint
	C.3.5 Factory production of plastic particles
	C.3.6 Toiletries
	C.3.7 Glitter
	C.3.8 Plastic trash
	C.3.9 Other
C.4 Where do you think microplastics are finally accumulating in the environment? (multiple choice)	
	C.4.1 Rivers and lakes
	C.4.2 Soil
	C.4.3 Air
	C.4.4 Animals and plants
	C.4.5 Ocean
	C.4.6 Beaches
	C.4.7 Human bodies
	C.4.8 Other
C.5 In which ways do you think microplastics might enter a human body? (multiple choice)	
	C.5.1 Water
	C.5.2 Honey
	C.5.3 Salt
	C.5.4 Seafood

	C.5.5 Breathing air
	C.5.6 Body scrub
	C.5.7 Cosmetics
	C.5.8 Other
C.6 What policies do you think the government should enact to reduce microplastic pollution?	
	C.6.1 Offer free reusable bags
	C.6.2 Award reusing plastic bags
	C.6.3 Increase taxes of plastic products
	C.6.4 Stop selling products with microplastics
	C.6.5 Education
	C.6.6 Strict plastic usage law
	C.6.7 Improve water purification technology
	C.6.8 Other
D Could you please rate the following statements? (between 1 = extremely unlikely / I totally disagree and 7= extremely likely / I totally agree)	
	D.1- Microplastics can cause serious problems in our society.
	D.2- Microplastics entering the body can be a serious danger to health.
	D.3- Microplastics destroy the marine ecosystem.
	D.4- I will reduce my consumption of everyday items containing microplastics.
	D.5- I will buy or use eco-friendly products whenever possible.
