# Supplementary material for: Greater willingness to reduce microplastics consumption in Mexico than in Spain supports the importance of legislation on the use of plastics
Source: Front Psychol. 2023 Jan 11;13:1027336. doi: 10.3389/fpsyg.2022.1027336 (PMC9875725; doi:10.3389/fpsyg.2022.1027336)
Supplement: Supplementary file 2 [file Table_2.pdf]

Supplementary table 2. Means (above) and their standard deviations (below) of the variables considered in this study, by country and discipline, for Mexican and Spanish students.

	Disciplines	Check microbeads	Reduce MP consumption	Use eco-friendly products	MP risk	Barriers	Known sources	Known sinks	Known body entries	Declared knowledge
Mexico	Agriculture	2,20	5,00	5,00	6,33	4,60	2,60	4,80	2,60	1,00
	Education	2,03	5,78	5,80	5,85	2,41	2,57	3,10	1,96	0,34
	General programmes	2,18	4,59	5,00	5,65	3,54	2,74	3,10	2,41	0,45
	Health	2,56	5,22	5,56	5,48	2,78	2,33	3,00	2,44	0,33
	Humanity & arts	1,75	4,92	4,67	4,86	2,42	2,42	2,08	2,58	0,29
	Science	2,00	4,86	6,57	5,95	3,86	2,00	2,71	1,57	0,40
	Services	2,30	5,80	5,70	6,40	4,00	3,30	2,90	3,10	0,29
	Social sciences	1,69	5,45	5,35	5,64	2,51	2,40	3,24	1,87	0,29
Spain	Agriculture	2,00	5,20	5,00	4,33	2,60	4,80	4,80	2,40	1,00
	Education	1,32	5,66	5,57	6,23	3,44	3,86	4,64	3,01	0,69
	General programmes	1,78	4,64	4,73	5,82	2,82	4,36	4,00	3,27	0,60
	Health	1,40	5,72	5,96	6,25	3,48	4,52	5,04	2,80	0,68
	Humanity & arts	1,67	4,56	5,00	5,44	3,22	3,56	4,89	2,33	0,89
	Science	1,75	4,90	4,80	4,92	3,10	4,90	5,00	2,60	0,85
	Services	1,09	4,55	4,64	6,06	3,09	2,82	3,73	3,00	0,55
	Social sciences	1,53	5,59	5,71	5,84	2,94	4,24	4,00	3,06	0,60
Mexico	Agriculture	1,10	2,35	2,35	0,71	1,52	2,07	1,79	1,52	0,00
	Education	1,04	1,83	1,80	1,72	1,40	1,74	1,86	1,30	0,48
	General programmes	1,19	2,14	2,01	1,85	1,80	1,86	1,97	1,50	0,51
	Health	1,33	2,44	1,13	1,66	1,09	1,41	1,58	1,33	0,52
	Humanity & arts	0,97	2,02	2,31	2,29	2,19	1,44	1,93	2,15	0,49

	Science	1,41	2,41	1,13	1,31	1,68	0,82	1,50	1,27	0,55
	Services	1,34	1,81	1,95	0,68	1,76	2,71	1,97	1,37	0,49
	Social sciences	0,90	2,06	2,07	1,88	1,55	1,36	1,92	1,11	0,46
Spain	Agriculture	1,22	2,05	2,00	1,70	2,07	3,19	2,39	2,07	0,00
	Education	0,68	1,48	1,58	1,14	1,69	2,32	2,10	1,65	0,46
	General programmes	1,39	1,69	2,05	1,83	2,27	3,53	3,38	2,83	0,52
	Health	0,65	1,51	1,57	1,26	1,92	2,50	2,07	1,76	0,48
	Humanity & arts	1,12	2,30	2,55	2,28	1,48	2,55	1,83	1,12	0,33
	Science	1,16	2,17	2,33	1,89	2,20	2,40	1,69	2,01	0,37
	Services	0,30	1,69	1,91	1,67	1,30	2,14	2,15	2,00	0,52
	Social sciences	0,94	1,80	1,57	1,73	1,78	3,07	2,47	2,51	0,51
